# Expanding the uses of genome‐scale models with protein structures

**DOI:** 10.15252/msb.20188601

**Published:** 2019-11-26

**Authors:** Nathan Mih, Bernhard O Palsson

**Affiliations:** ^1^ Department of Bioengineering University of California San Diego La Jolla CA USA; ^2^ Bioinformatics and Systems Biology Program University of California San Diego La Jolla CA USA; ^3^ The Novo Nordisk Foundation Center for Biosustainability Technical University of Denmark Lyngby Denmark

## Abstract

Biology is reaching a convergence point of its historic reductionist and modern holistic approaches to understanding the living system. Structural biology has historically taken the reductionist approach to deeply probe the inner workings of complex molecular machines. In contrast, systems biology and genome‐scale modeling have organically grown out of the wealth of data now being generated by diverse omics measurements. In the late 2000s, a proposed interdisciplinary field of structural systems biology pitched the merger of these two approaches, with widespread applications in pharmacology, disease modeling, protein engineering, and evolutionary studies. In this commentary, we highlight the challenges of integrating these two fields, with a focus on genome‐scale metabolic modeling, and the novel findings that are made possible from such a merger.

## Abstract 2

It is now possible to mechanistically integrate two separate large fields in biology: systems and structural biology. We explore how this integration has enabled new research directions in metabolic modeling and beyond.

## Graphical abstract

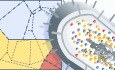

## A challenge for converging fields

The field of structural systems biology represents an integration of two established, but quite different, fields: structural biology and systems biology. Given the different histories, underlying paradigms, ways of thinking, and the characteristics of the data types used in these two fields, such an integration is not without its challenges. In spite of these fundamental differences, a convergence is not only happening, but is necessary to achieve the ultimate goals of systems biology.

The number of experimental structures in the Protein Data Bank (PDB) continues to steadily rise each year. A key distinction between structural data and the omics data types prevalent in systems biology is the capability of structural data to “zoom in” to the atomic level to study fundamental details of chemical interactions. A structural biologist knows the value of mechanistic insights that can be gained from this information. Structural data offer new features, such as a three‐dimensional context to mutations, post‐translational modifications, protein domains, linking needs for functionalizing prosthetic groups to metabolism, ROS damage sites, and others, enabling the execution of novel studies in systems biology. We have now reached the point where structural information for certain organisms, such as *Escherichia coli* and *Homo sapiens*, can be utilized at the systems level.

In contrast to structural biology, a systems biologist “zooms out” to see thousands of biomolecular interactions happening simultaneously. The totality of such interactions is experimentally studied through the generation of various omics data types and by constructing large‐scale mechanistic frameworks to relate individual components represented in such data sets. The success of genome‐scale metabolic modeling can be attributed to high‐quality, bottom‐up reconstructions of metabolic, protein synthesis, and transcriptional regulatory networks on an organism‐specific basis.

## What does systems biology need from structural biology?

To understand how structural biology can be utilized like an additional omics data source in systems biology, we first describe how we use the terms “structural genomics” and “structural proteomics”. Structural genomics has widely been used to describe the determination of all 3D structures of proteins within an organism's genome. Worldwide collaborations have led to the deposition of over 150,000 structures in the PDB, and novel protein folds and families have been uncovered as a result of this exhaustive effort. With systems biology models, there is a clear benefit to having this information available, making accessible a literal new dimension of information to describe the components of a cell down to the molecular level.

However, knowing just the 3D structures of proteins is only one‐half of what is needed to fully realize structural data as an omics data type. The term structural proteomics has increasingly been used to describe novel experimental approaches for determining the millions of transient interactions between proteins and other components of the cell (Piazza *et al*, 2018). Thus, we can attempt to delineate the two terms, with one being defined as the study of what is encoded and produced directly from the genome (structural genomics) and the other as the result of these encoded components interacting with their environment (structural proteomics). Yet, there is no clear separation between the two terms as structural determination technologies advance—in particular, in‐cell NMR techniques actually resolve structures at an atomic level within a cell (Tanaka *et al*, 2019), while cryo‐electron tomography (cryo‐ET) promises to answer both questions of structures and interactions by visualizing all components interacting with each other at a point in time (Beck & Baumeister, 2016). For clarity, in this commentary we will simply refer to the overall collection of protein structures in the context of systems models as the structural proteome.

With these data sets becoming commonly available, new computational and data challenges arise. Computational tools are required for numerous integration problems, such as for putting together the pieces of higher‐order protein complexes, filling in the gaps of missing structures and interactions with *in silico* predictions, and formally integrating data derived from this information within systems models. None of these are trivial problems to solve, but recent advances in all these areas encourage us that this future is not too far away.

## Adding protein structures to genome‐scale metabolic models

**Figure 1:**
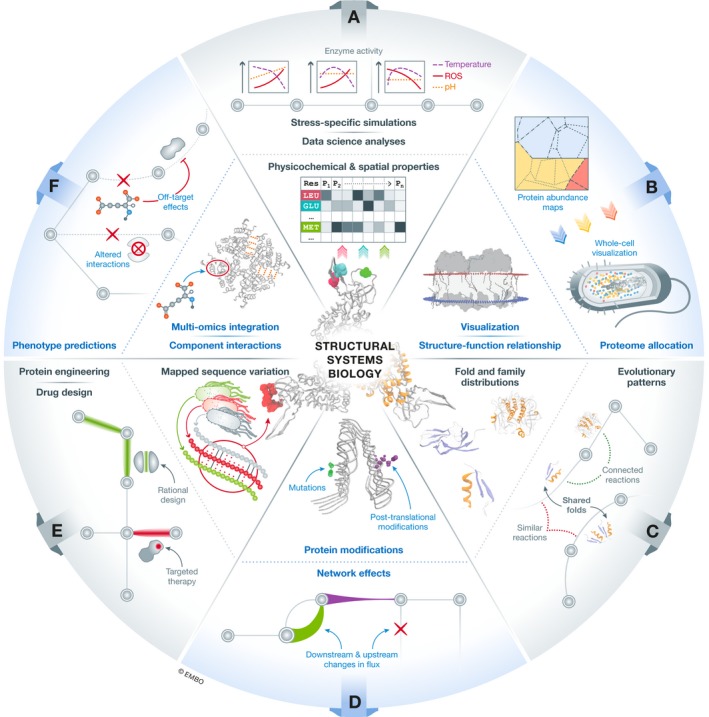
Classification of structural systems biology studies into six use categories (A) Detailed physicochemical and spatial properties of the structural proteome enable the use of protein structures as an “omics” data source. This additional information has been utilized for downstream data science analyses and advanced metabolic modeling simulations incorporating residue‐level measurements for applications such as stress‐specific simulations. (B) A better understanding of functional assignments of proteins based on their structures leads to improved genome‐scale models, which better predict protein abundances in different conditions. Currently available structural determination techniques lead to visualizations of intricate protein complexes and their place within the cell, leading to potential realistic models of whole cells that reflect these environmental conditions. (C) Classical analyses of protein fold usage in the context of metabolic networks add an additional level of functional understanding to the network. The analysis of how folds are distributed within a network, and between strains or species, answers questions about the patterns we observe in metabolic pathway evolution. (D) *In silico* molecular modeling tools enable residue‐level predictions of mutations or post‐translational modifications that can then be used to modulate changes within a metabolic network, leading to an understanding of the global effect of small changes upon an entire cell. (E) Large‐scale analyses of sequence variation mapped to structure can uncover how small differences in protein structure potentially lead to metabolic changes within different strains of an organism. Furthermore, these small differences are crucial to finding regions in proteins that may cause undesirable pharmacogenomic interactions and can be crucial in the drug design process. As an extension of this, enzyme engineers can utilize this information to understand where highly variable regions of proteins are, to design more targeted libraries in the engineering cycle. (F) The interactions between proteins and the other components of the cell have only now been characterized at a large scale. Previously, interactions such as protein–ligand binding events have largely been limited to those directly involved in catalysis and studied by enzymatic assays. A large‐scale understanding of all small molecule interactions with proteins inside a cell adds yet another “interactome” to systems biology models, uncovering competitive and non‐competitive interactions that regulate processes alongside the transcriptome. Beyond these, protein–protein, protein–DNA, and gene–metabolome interaction data sets all contribute to our better understanding of the cell, but require a scaffold on which data need to be mapped.

In 2009, a structural genomics project for the thermophile *Thermotoga maritima* was completed. At the same time, a newly reconstructed metabolic network of *T. maritima* provided the context with which to analyze this compendium of protein structures (Zhang *et al*, 2009). This pioneering study created the first genome‐scale model with protein structures, or GEM‐PRO. With this integrated model, the authors addressed questions associated with the evolution of new pathways in a metabolic network, focusing on two competing theories of pathway evolution. One theory posited that functionality evolves by the recruitment of nearby neighbors in a metabolic pathway that are likely to carry out similar enzymatic changes. Another theory stated that functionality evolved through the recruitment of promiscuous enzymes from faraway parts of a metabolic network to carry out the next steps of a pathway. By looking at the protein folds in the context of a network, it was found that folds were quite different compared to neighboring enzymes, leading to the conclusion that functionality likely evolved through the second, promiscuous recruitment model (Fig 1C). This study demonstrated that a synergy between structures and their location in a network was required to answer an evolutionary question. If the structures were the only source of information available, it would not be possible to understand their position within a metabolic network. At the same time, with just a metabolic network, no knowledge of the protein folds in 3D space would be available.

The integration of structural data into metabolic models has subsequently been extended to a number of applications, which we categorize and review below.

### The composition, biosynthesis, and visualization of proteins

Similar to how genome content can be summarized by their nucleotide building blocks, the structural proteome of an organism can be described in terms of the biochemistry of its proteins and their amino acid composition. Simple physicochemical properties (polarity, hydrophobicity, size) can act as descriptors, and more detailed spatial information such as location and function can be included—given a characterized 3D structure of the protein (Fig 1A). These data can be prepared for downstream data science analyses and can further enhance modeling capabilities by introducing quantitative predictions of enzyme activity based on these properties at different stress conditions.

How does a cell determine its allocation of resources to the variety of machinery that drives its growth and maintains its functions? A new generation of genome‐scale metabolic models incorporate the cost of synthesizing the enzymes that carry out metabolic reactions in a cell. Enzyme turnover rates then become crucial pieces of information that need to be curated or estimated for all of a model's reactions. Recently, we turned to structural information to aid in the prediction of these notoriously hard to estimate rates, using machine learning methods for a proteome synthesis model of *E. coli* (Heckmann *et al*, 2018). Structural features describing the active sites of these enzymes and general descriptors of the global protein structure were incorporated alongside a number of other features into the predictive model.

The structure–function relationship also provides fundamental information for describing an enzyme's role within a metabolic network. As more and more structures are solved, similarities between them and well‐studied proteins allow us to confidently assume functionalities when reconstructing metabolic networks. As we approach the completion of these features, large‐scale visual representation becomes possible. What may be viewed as the “final frontier” of structural systems biology would be an accurate visual 3D model of a cell, incorporating all known molecular interactions, localizations, abundances, enzyme complex stoichiometries, higher‐order DNA structures, small molecules, and more (Fig 1B). New work with cryo‐electron tomography has begun to uncover the assemblies of higher‐order protein complexes in their native environment (Beck & Baumeister, 2016). This promises to provide a visual connection between proteomics measurements and individually solved structures. Furthermore, it is now possible to obtain atomic‐level NMR structures of enzymes in these native environments—the final piece of the puzzle needed to view the components of a cell in their natural state, *in vivo* (Tanaka *et al*, 2019). Systems biology models provide the platform onto which these types of data can be mapped and represented computationally.

A predictive and visual model of proteome allocations under different conditions remains a challenge, as it is no small undertaking to manually gather and reconstruct enzymatic pathways, let alone simulate these models of growing complexity.

### The big picture of small changes

How do protein changes at the residue level impact the metabolic network and phenotypic behavior as a whole? Can the totality of these changes help classify cell types in humans or delineate between species and strains of unicellular organisms?

As a proof‐of‐concept, molecular modeling tools were used to analyze the impact of coding mutations on drug binding in the human red blood cell (Fig 1D; Mih *et al*, 2016). Docking and molecular dynamics simulations enabled predictions of differences in the binding affinities of small molecules due to a mutation in selected proteins. These relative differences were integrated into both constraint‐based and kinetic metabolic models of the red blood cell to observe the predicted systemic impact of the mutation upon metabolism. Sequence variation can also be mapped from different strains of a species, different species altogether, or from different cell lines (Fig 1E). Combined with spatial descriptors of amino acids, this approach can pinpoint certain protein domains with causal mutations that cluster together. The sequence variation of G‐protein‐coupled receptors (GPCRs) is a likely reason behind adverse effects that are observed in a minority of individuals who take a certain drug (Hauser *et al*, 2018). A comprehensive effort to map this variation from almost 70,000 individuals revealed numerous variants that appear close to drug binding pockets or other functional sites on GPCRs. Certain drug labels already include such warnings for characterized variants if an adverse reaction is known to occur, and this personalized information can only improve as more interaction data are gathered.

Extending these approaches to new methods such as those that attempt to model strain‐specific metabolism, we can begin to simulate differences at the resolution of single amino acids. Machine learning algorithms can take advantage of this information to identify patterns of genetic changes due to certain stresses, such as drug‐resistant strains of pathogens, and be used to develop predictive models for newly sequenced strains.

### The new era of multi‐omics data

The study of interactions between proteins and small molecules has largely been limited to those directly related to catalysis, studied with enzymatic assays. A large‐scale understanding of all small molecule interactions with proteins inside a cell adds yet another interactome to systems biology models, uncovering competitive and non‐competitive interactions that regulate processes alongside the transcriptome (Fig 1F).

Early work in this area focused on predicting the effects of small molecules binding to undesired targets, also known as off‐targets. A human kidney metabolic model integrated with structural information was used to reveal the potential mechanism of action of torcetrapib, a drug that was in development to treat high cholesterol levels (Chang *et al*, 2010). Retrospectively, this approach was utilized to give potential explanations as to why the drug gave rise to fatal hypertension in patients during clinical trials. This study applied binding site similarity predictions to human protein structures to identify potential targets that this drug could inhibit in addition to its main target. By combining these predictions with gene knockouts in a metabolic model of the human kidney, the authors were able to predict the clinically observed hypertension as a potential side effect of these off‐target binding events.

Experimental data sets representing these transient interactions with protein molecules are beginning to take shape. For the metabolome, ingenious techniques that apply proteolytic and mass spectrometric methods mean that protein–small molecule interactions can be elucidated at a scale comparable to other omics methods (Piazza *et al*, 2018). An additional layer of information can be added by measuring the effects of gene deletions on metabolite levels, in what are known as gene–metabolome data sets (Fuhrer *et al*, 2017). For the other large molecules within the cell, a number of biomolecular interaction screens are available and should be next in line to be explicitly accounted for in structural systems biology models. These include protein–protein interaction techniques and data sets, which not only provide information about protein complex compositions but also introduce cell signaling into the mix. The DNA interactome can be deciphered through protein–DNA interactions as measured by methods such as ChIP‐exo and predicted with newly developed algorithms that take into account the 3D structure of DNA motifs (Chiu *et al*, 2017).

Taking all of this information together and distilling it into a snapshot of a cell at work is perhaps the biggest challenge of them all, as data sets balloon in size when considering the billions, perhaps trillions, of interactions—all occurring at once.

## What lies ahead for structural systems biology?

The number of studies presented here demonstrates the growing range of applications enabled by the inclusion of protein structures into network models of metabolism, proteome synthesis, and proteostasis. We can now comfortably state that the structural proteome is now an available independent omics data type that (i) can be assembled for model organisms, (ii) can be computationally represented and integrated into genome‐scale models, and (iii) adds new dimensions to systems biology studies where a fundamentally new set of questions can be addressed. Although we are still far from realizing a fully integrated multi‐scale whole‐cell model, these studies show that even with incomplete information, the continued development and application of structural systems biology leads to novel applications.

## Conflict of interest

The authors declare that they have no conflict of interest.

## References

[msb188601-bib-0001] Beck M , Baumeister W (2016) Cryo‐electron tomography: can it reveal the molecular sociology of cells in atomic detail? Trends Cell Biol 26: 825–837 27671779 10.1016/j.tcb.2016.08.006

[msb188601-bib-0002] Chang RL , Xie L , Xie L , Bourne PE , Palsson BØ (2010) Drug off‐target effects predicted using structural analysis in the context of a metabolic network model. PLoS Comput Biol 6: e1000938 20957118 10.1371/journal.pcbi.1000938 PMC2950675

[msb188601-bib-0003] Chiu T‐P , Rao S , Mann RS , Honig B , Rohs R (2017) Genome‐wide prediction of minor‐groove electrostatic potential enables biophysical modeling of protein‐DNA binding. Nucleic Acids Res 45: 12565–12576 29040720 10.1093/nar/gkx915 PMC5716191

[msb188601-bib-0004] Fuhrer T , Zampieri M , Sévin DC , Sauer U , Zamboni N (2017) Genomewide landscape of gene‐metabolome associations in *Escherichia coli* . Mol Syst Biol 13: 907 28093455 10.15252/msb.20167150 PMC5293155

[msb188601-bib-0005] Hauser AS , Chavali S , Masuho I , Jahn LJ , Martemyanov KA , Gloriam DE , Babu MM (2018) Pharmacogenomics of GPCR drug targets. Cell 172: 41–54.e19 29249361 10.1016/j.cell.2017.11.033 PMC5766829

[msb188601-bib-0006] Heckmann D , Lloyd CJ , Mih N , Ha Y , Zielinski DC , Haiman ZB , Desouki AA , Lercher MJ , Palsson BO (2018) Machine learning applied to enzyme turnover numbers reveals protein structural correlates and improves metabolic models. Nat Commun 9: 5252 30531987 10.1038/s41467-018-07652-6 PMC6286351

[msb188601-bib-0007] Mih N , Brunk E , Bordbar A , Palsson BO (2016) A multi‐scale computational platform to mechanistically assess the effect of genetic variation on drug responses in human erythrocyte metabolism. PLoS Comput Biol 12: e1005039 27467583 10.1371/journal.pcbi.1005039 PMC4965186

[msb188601-bib-0008] Piazza I , Kochanowski K , Cappelletti V , Fuhrer T , Noor E , Sauer U , Picotti P (2018) A map of protein‐metabolite interactions reveals principles of chemical communication. Cell 172: 358–372.e23 29307493 10.1016/j.cell.2017.12.006

[msb188601-bib-0009] Tanaka T , Ikeya T , Kamoshida H , Suemoto Y , Mishima M , Shirakawa M , Güntert P , Ito Y (2019) High resolution protein 3D structure determination in living eukaryotic cells. Angew Chem Int Ed Engl 58: 7284–7288 30938016 10.1002/anie.201900840

[msb188601-bib-0010] Zhang Y , Thiele I , Weekes D , Li Z , Jaroszewski L , Ginalski K , Deacon AM , Wooley J , Lesley SA , Wilson IA *et al* (2009) Three‐dimensional structural view of the central metabolic network of *Thermotoga maritima* . Science 325: 1544–1549 19762644 10.1126/science.1174671 PMC2833182

